# Emerging Picture of Deuterosome-Dependent Centriole Amplification in MCCs

**DOI:** 10.3390/cells7100152

**Published:** 2018-09-27

**Authors:** Umama Shahid, Priyanka Singh

**Affiliations:** Department of Bioscience & Bioengineering, Indian Institute of Technology Jodhpur, NH 65, Nagour Road, Karwar 342037, India; shahid.1@iitj.ac.in

**Keywords:** cilia, centriole, deuterosome, multiciliated cells, basal body

## Abstract

Multiciliated cells (MCCs) have several hair-like structures called cilia, which are required to propel substances on their surface. A cilium is organized from a basal body which resembles a hollow microtubule structure called a centriole. In terminally differentiated MCCs, hundreds of new basal bodies/centrioles are formed via two parallel pathways: the centriole- and deuterosome-dependent pathways. The deuterosome-dependent pathway is also referred to as “de novo” because unlike the centriole-dependent pathway which requires pre-existing centrioles, in the de novo pathway multiple new centrioles are organized around non-microtubule structures called deuterosomes. In the last five years, some deuterosome-specific markers have been identified and concurrent advancements in the super-resolution techniques have significantly contributed to gaining insights about the major stages of centriole amplification during ciliogenesis. Altogether, a new picture is emerging which also challenges the previous notion that deuterosome pathway is de novo. This review is primarily focused on studies that have contributed towards the better understanding of deuterosome-dependent centriole amplification and presents a developing model about the major stages identified during this process.

## 1. Introduction

Centrosomes act as major microtubule organizing centers in the majority of animal cells, forming a bipolar spindle during cell division. Despite being identified and described by Van Beneden [1] and Theodore Boveri [2] in the late 19th century, centrosomes did not get much attention for a long period, probably due to the fact that several cell types can still divide without any visible centrosome structure [3]. Centrosomes were later identified to be involved in a plethora of cell functions such as cell migration [4], cell signaling [5], ciliogenesis [6] and brain development [7]. From the mid to late 20th century, centrosomes gained much deserved attention due to the growing evidence correlating them to several human diseases. For instance, supernumerary centrosomes were observed in several human cancer patients [8,9]. Furthermore, disruption of centrosome-associated proteins has been linked to brain disorders such as autosomal recessive primary microcephaly (MCPH) [10]. With advances in the field of proteomics and microscopy [11], our understanding of the structural organization of centrosomes and the molecular players involved in its biogenesis has significantly improved.

Electron microscopy revealed that the core of a centrosome consists of two orthogonally arranged hollow cylindrical microtubule structures called centrioles, surrounded by a proteinaceous cloud called pericentriole material (PCM) [12]. A cell usually contains one centrosome which replicates by duplicating its core structure i.e., centrioles, during the S phase of the cell cycle. Centriole duplication is template-dependent, wherein the pre-existing centriole pair separates and each centriole then acts as a template for the organization of a new cartwheel-like structure called procentriole. The procentriole elongates or matures adjacent to the pre-existing centriole. The two centrosomes thus formed move towards two ends of a cell where each of them functions as a pole for bipolar spindle organization during the M phase of a cell cycle. After the cell division, each daughter cell receives one centrosome [13].

In certain cell types, centrioles can also organize “de novo”, that is without the need for any template. The de novo origin of centrioles has been described in parthenogenetically developing insects [14,15], mammalian multiciliated cells (MCCs) [16,17,18], and in unfertilized *Drosophila* eggs overexpressing core centriole proteins [19,20]. Laser ablation of pre-existing centrioles in somatic cells results into the generation of new centrioles via the de novo centriole pathway. In these cells, the de novo centriole organization was found to be significantly slow as compared to the template pathway [21,22]. In contrast to the template-dependent pathway which strictly regulates centriole numbers per cell, the de novo pathway results in error prone centriole structures and a random increase in centriole number [23]. Sudden boost in centriole number (hundreds) is quite apparent in MCCs during ciliogenesis. Centriole amplification in MCCs is mostly a result of the deuterosome-dependent (DD) pathway. Deuterosomes are non-microtubule fibrous bodies around which procentrioles are organized [17,18,24]. Since the DD pathway does not involve any centriole-like structure as a template, it is also referred to as “de novo”. Several classical early centriole markers are identified on centrioles generated by the DD pathway, indicating that these centrioles also preserve classic procentriole features. Some deuterosome-specific proteins like Deup1 [25], CCDC78 [26] and CCNO [27] have been identified in the last five years, suggesting that the DD pathway is also quite regulated. These growing evidence mostly acquired through super-resolution microscopy and live imaging in MCCs instigates an interesting correlation between pre-existing centrioles and deuterosome organization [28]. We are at a juncture where a new picture of DD pathway is shaping up and, accordingly, the present review summarizes all these studies which have contributed to the changing picture of the DD pathway in MCCs.

## 2. Centriole Structure and Organization

**Figure 1:**
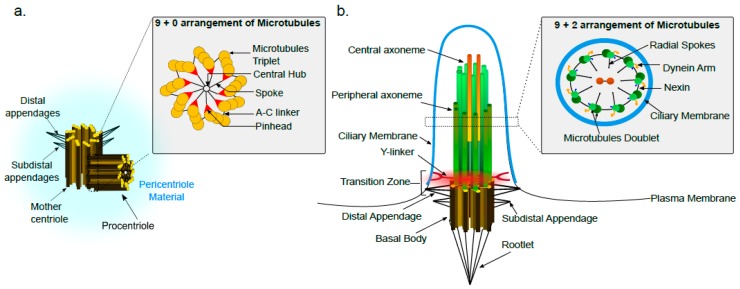
Schematic representation of (**a**) centrosome highlighting the 9 + 0 arrangement of triplet microtubules (box) and (**b**) a cilium showing 9 + 2 arrangement (motile cilium) of doublet microtubules (box). The major features of the centrosome/cilium are marked. The mother centriole triplets are extended into cilia doublets. The C-tubule extends into the transition zone of the cilium which is also decorated by the Y-fibers.

A centriole is a barrel shaped structure exhibiting nine-fold symmetric arrangement of triplet microtubules, A-, B- and C-tubules from internal to external, are arranged with an anticlockwise twist (Figure 1a). The A-tubule is consisting of 13 α- and β-tubulin containing protofilaments, whereas B- and C-tubules have 10 protofilaments successively arranged to form the triplet [12]. Cryoelectron tomography and cross sectional images have revealed that there are additional non-tubulin protein densities between the A- and C- tubules acting as a linkers and presumably required for centriole structure stability [29]. The centriole structure is evolutionarily conserved, although some structural variations can be observed. For example, somatic cells of *Drosophila* have doublets [30], whereas *Caenorhabditis elegans* has singlet microtubules in their centrioles [31]. Although, the centriole structure has been reported to be constant in different tissues of an organism, some exceptions to this rule exist. For instance, the fungus *Sciara* has giant centrioles containing 60–90 singlet microtubules in their germ line tissue, however the centrioles found in the *Sciara* somatic cells have classic 9 fold symmetric arrangement of triplet microtubules [32]. Similarly, centrioles in *Drosophila* germ line stem cells contains nine triplet microtubules whereas nine doublet microtubules containing centrioles are observed in *Drosophila* somatic cells [30]. The functional relevance of triplet versus doublet arrangement of microtubules in centrioles is not entirely clear. Although, it has been observed that *uncoordinated* (*unc*) *Drosophila* mutants lacking C-tubules have severely affected centriole growth and axoneme formation which in turn affects ciliary projection assemblies in these cells [33].

Vertebrate centrioles measure roughly 200 nm in diameter and 500 nm in length. The centriole pair in a centrosome is asymmetric, the more mature centriole is referred as the “mother” centriole, whereas the less mature one is called the “daughter” centriole. The mother centriole is slightly longer than the daughter centriole and in vertebrates it has been shown to have some specific proteinaceous features like distal and sub-distal appendages [12]. Due to the small size of centrioles which falls at the border of the diffraction limit of light microscopy, our major understanding about centriole structure was previously derived from electron microscopy techniques. However, recent advances in super-resolution and fluorescence microscopy methods have contributed tremendously to a better understanding about the centriole structure organization and dynamics [34].

Often centriole biogenesis is compared to DNA replication because of its need for a template to assemble the new centriole. At the late M/G1 stage of the cell cycle, the mother and daughter centrioles separate slightly but remain held together by a proteinaceous linker. A new centriole called a procentriole is formed orthogonally to each parent centriole during the S phase of the cell cycle. Procentrioles are cartwheel-like structures, containing nine spokes extending from the center of the cartwheel towards peripheral microtubules [13]. Structural studies have been quite useful in identifying the role of SAS6 protein in dictating the nine-fold symmetry of centriole microtubules [35,36]. Human centrosomes consist of hundreds of proteins as revealed by their proteomic characterization [37]. Model organisms like *C. elegans* [38] and *Drosophila melanogaster* [39] have been very useful in providing an understanding about the protein dynamics during the centriole duplication pathway. The core centriole proteins work in a hierarchical fashion during cartwheel formation and many of these proteins are found to be evolutionarily conserved [13]. The CEP63 protein is localized to the proximal end of the mother centriole and interacts with ring-like structure formed by CEP152 [40]. CEP152 together with CEP192 recruits the auto regulatory master player Polo-like Kinase4 (PLK4) [41]. PLK4 binds and phosphorylates SCL/TAL1-interrupting locus (STIL) protein at its C-terminus conserved region, called the STAN domain. Blocking STAN domain phosphorylation abolishes STIL and SAS6 interaction, suggesting that the STAN domain phosphorylation is required for the binding and recruitment of the cartwheel protein SAS6 [42]. STIL is also found to interact with another important centrosome protein, CENPJ/CPAP, which triggers the growth of microtubules and controls the length of new centrioles [43,44,45,46]. Centriole number is regulated by controlling levels of core centriole proteins. Overexpression of the core centriole proteins PLK4 [47], SAS6 [48] and STIL [49,50] results in the formation of a “rosette” of new centrioles around parent centrioles, whilst their down regulation results either in fewer centrioles or defects in the centriole structure.

## 3. The Base of Cilia: Basal Body

In several resting cells and certain terminally differentiated cells, centrioles form cilia. Cilia are hair-like structures with a microtubule skeleton referred as axonemes, which in turn consist of doublet microtubules arranged in a pinwheel fashion and surrounded by the ciliary membrane (Figure 1b). Cilia can be motile or non-motile, differing at their structural level. For instance, motile cilia contain 9 outer and 2 central doublets (9 + 2 arrangement) but non-motile cilia have only 9 outer doublets (9 + 0 arrangement) [51,52,53]. At the time of ciliogenesis, the mother centriole of the centrosome is positioned very close to the plasma membrane and the ciliary microtubules are nucleated from its distal end by virtue of the distal appendages [54]. The mother centriole involved in cilium formation is now referred to as the basal body. Although the basal body has a triplet arrangement of microtubules, after the transition zone in the cilium, doublet microtubules extending from the basal body are observed. The peripheral ciliary axonemes are in continuation with the A- and B-tubules of the basal body. The C-tubule of the basal body reaches only till the transition zone of cilia [55]. In the transition zone, Y-shaped fibers connect the microtubules to the ciliary membrane [56]. These Y-shaped fibers acts as a ciliary gate which is involved in regulating intraflagellar transport (IFT) [57]. Although, the central axonemes of cilia are not continuous with the basal body, inhibition of the basal body protein BLD10/CEP135 leads to defects in central axoneme formation [58]. Other classical features of the basal body include the rootlet, distal appendages and sub-distal appendages/basal foot. Using quantitative proteomics and super-resolution microscopy, at least five distal appendage proteins (CEP164, CEP89, CEP83, SCLT1 and FBF1) have been identified. All these proteins are found to be important for ciliogenesis and among them specific blocking of CEP83 protein affects centriole membrane docking [59]. A recent report has identified novel proteins on sub-distal appendages [60] but the role of these newly identified proteins as well as of the sub-distal appendages in ciliogenesis need further investigation. The ciliary rootlet projecting from the proximal end of the basal body is a thick striated bundle of filaments and proposed to be required for the long term stability of sensory cilia [61]. Accordingly, studies on *Drosophila melanogaster* Rootletin (Root), an orthologue of the mammalian Rootletin, showed that root mutant neurons lack rootlets and have dramatically impaired sensory function. These root mutant flies showed mechano-sensation and chemo-sensation behavioral defects [62].

Most cells have a single non-motile primary cilium, a site for several important cell signaling events such as sonic hedgehog (shh) and Wnt signaling that are involved in transducing any chemical and mechanical changes from the extracellular environment of the cell to its interior [5]. Ciliated protists [63,64] and flagellated sperms of lower plants [65] contain multiple motile cilia. Animal cells of the respiratory tract [66], oviducts [16] and the ventricular cells of the brain [67] also contain multiple cilia. These cells are collectively referred to as multiciliated cells (MCCs). In these cells, cilia assist in propelling substances like air, eggs, fluids, mucus, particles or pathogens over the epithelia surface by virtue of coordinated beating. Any genetic defect that disrupts cilia functions result in a number of human diseases called ciliopathies [68]. Contrary to restricted centriole biogenesis in cycling cells, hundreds of centrioles are generated during ciliogenesis in MCCs, which eventually migrates apically to generate a basal body anchoring a motile cilium [17,18,24]. Although similar looking microtubule structures are visible during the centriole biogenesis in cycling cells and centriole amplification in ciliogenesis, the following basic differences exist: (1) centriole biogenesis is restricted to the organization of only one new centriole adjacent to the pre-existing centriole, whereas during ciliogenesis in MCCs multiple centrioles or rosettes of centrioles are observed; (2) centriole biogenesis in cycling cells is a mitotic event whereas centriole amplification during ciliogenesis happens in terminally differentiated (post-mitotic) cells. However, a recent report has shown that the mitotic machinery is transiently active during centriole amplification to centriole disengagement stages of these post-mitotic multiciliated progenitors [69]. Furthermore, the vertebrate-specific cell division cycle 20B (CDC20B) protein is found to be associated with deuterosomes and it has been shown to be required for the production of centrioles and cilia in mouse and *Xenopus* MCCs [70].

## 4. Centriole Amplification Pathways in Multiciliated Cells (MCCs): Centriole- and Deuterosome-Dependent Pathways

Serial sectioning, stereomicroscopy, and image-enhanced photography techniques were employed in rhesus monkey oviducts to show that multiple centrioles in ciliated cells arise via two parallel pathways, namely (1) the centriole-dependent (CD) pathway, which contributes to 5% of centriole population in ciliated cells and during this pathway multiple centrioles are organized around the mother centrioles (rosettes of centrioles); (2) the deuterosome-dependent (DD) pathway, which is responsible for 95% of the centriole population in ciliated cells. The DD pathway is also called the “de novo” pathway since new centrioles are organized around the deuterosomes, the fibrous non microtubule structures [71]. The molecular mechanisms underlying the DD pathway are not clear, but several reports have emerged that shed light on the important steps in this process. 

The CD pathway resembles the overexpression phenotype of core centriole proteins like PLK4 [47], SAS6 [48] and STIL [49,50], resulting in rosettes of procentrioles adjacent to the pre-existing centrioles. The de novo centriole pathway is evolutionarily conserved as it is present in lower organisms like multiflagellated germ cells in termites [72] and molluscs [73]; several parthenogenetically developing insects like *Muscidifurax uniraptor* [74] and stick insects of the genus *Bacillus* [75]; during gametogenesis in lower plants and fungi [76,77] like *Spisula* [78], *Naegleria* [79] and in *Oxytricha* [80], where centrioles appear from many foci of microtubules during the first cell division. The de novo pathway is also observed during the artificial activation of parthenogenesis in several species like *D. melanogaster* [15], sea urchins [81] and rabbits [82]. Laser ablation of primary centrioles in cells like CHO, HeLa [22] or human telomerase reverse transcriptase (hTERT)-expressing cells during the S phase also results in activation of the de novo centriole pathway [21]. Although it is not explicit whether the pre-existing centrioles play any role in the de novo centriole amplification pathway in many cell types, there is a growing body of evidence from mammalian MCCs, suggesting that the DD centrioles amplification in these cells could not just simply be considered as de novo.

## 5. Changing Picture of Deuterosome-Dependent (DD) Pathway

In 1969, Kalnins and Porter [17] described centriole replication in tracheal epithelial cells of 15- to 19-day-old chick embryos during ciliogenesis. Electron microscopy revealed that some procentriole clusters are directly attached to the pre-existing centriole. The authors also reported fibrous regions around the pre-existing centrioles which are involved in centriole biogenesis, thereby providing the notion that the pre-existing centrioles might play some role in the organization of these fibrous regions during ciliogenesis.

The in vitro differentiation model of mouse tracheal epithelia cells (MTECs) has been very useful in understanding centrosome amplification during ciliogenesis at the air–liquid interface (ALI). Stearns and colleagues [83] used fluorescently tagged centriole protein markers i.e., CEP135 and acetylated α-tubulin to identify the four stages during centriole amplification in MTECs. They reported the localization of CEP135 foci near the centrosomes at the apical cytoplasm of MTECs during stage I. This was followed by CEP135 cluster formation at one side of the cell, which the authors referred to as stage II. In stage III, CEP135 cluster gets dispersed in the cell and later appears near the plasma membrane. The authors reported the appearance of the evenly distributed basal bodies on the plasma membrane with anchored cilia at the final stage IV. This work contributed to the initial understanding of centriole amplification dynamics during ciliogenesis in MCCs.

In 2013, Zhao et al. [25] and Klos Dehring et al. [26] published two independent research articles, where they reported the identification of specific deuterosome markers, Deup1 (also referred as Ccdc67) and CCDC78, respectively.

**Figure 2:**
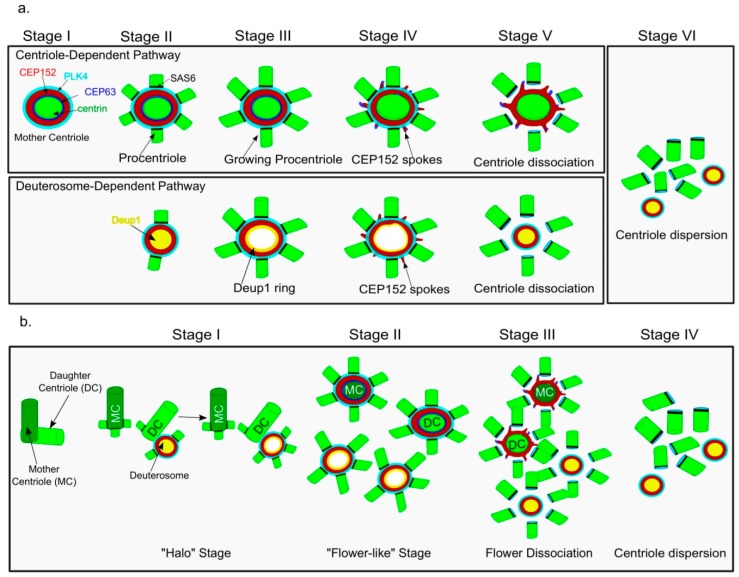
Comparison of the early stages of centriole amplification in MCCs as reported by (**a**) Zhao et al., 2013 and (**b**) Al Jord et al., 2014. The figure is adapted and modified from their respective papers for simplicity. Procentrioles are visible using Centrin as a marker. The stage I reported in Al Jord et al. corresponds to the stages II and III reported by Zhao et al., 2013 where procentrioles decorate deuterosomes, which are organized adjacent to the pre-existing daughter centriole (reported by Al Jord et al., 2014). Note that the localization of Deup1 is reported to change from being observed as foci inside the CEP152 ring to attaining a ring-like structure itself. In the later stages, procentrioles mature on their respective template-either the pre-existing centriole or deuterosome, to assume a flower-like arrangement. Subsequently, they are dispersed in the cytoplasm, only to reach at the apical side of membrane, generating ciliary tufts.

Zhao et al. used bioinformatics to identify Deup1 as a paralogue of the centriole protein CEP63. Phylogenetic tree analysis indicated that Deup1 duplicated and diverged from CEP63 during vertebrate evolution. Deup1 mRNA was found to be highly enriched in multiciliated tissues like the trachea and oviducts. Just like its paralogue CEP63 [40], Deup1 was also found to interact with CEP152 via its C-terminus region. The ectopic expression of Deup1 in U2OS cells resulted into deuterosome-like structures as observed by 3D structured illumination microscopy (3D-SIM). Experiments done using in vitro differentiated MTECs revealed that Deup1 and CEP63 protein levels get elevated during ciliogenesis together with other centriole markers like PLK4, CEP152, CPAP, SAS6 and STIL. The authors reported that Deup1 protein levels are 7.3 fold higher as compared to CEP63 at day 3 of ALI, which indicates a dominant role of the DD pathway in centriole production during ciliogenesis. Super-resolution 3D-SIM images of these cells revealed that Deup1 is mostly localized at deuterosomes. Depletion of Deup1 in MTECs resulted in repressed DD centriole amplification but enhancement of CD centriole amplification, suggesting some cross-talk between these two pathways. Zhao et al. used 3D-SIM to describe the six stages of centriole amplification in MTECs at day 3 of ALI (Figure 2a). They observed that centriole amplification starts from stage II, where Deup1 foci was localized inside the ring of CEP152 and this ring was found to be decorated with several centrin-positive (early centriole marker) dots. In the case of stage III, Deup1 appeared as a ring shaped structure with procentrioles arranged at the outer part of the ring, thereby resembling the flower-like arrangement. During stage IV, spoke-like structures were visible at the outer edge of CEP152 ring formed during both the CD and DD mediated centriole amplification pathways. In stage V, the new centrioles organized around the mother centriole as well as the deuterosomes are dispersed from their respective cradle. In the final stage VI, multiple centriole clusters with no visible association with their respective cradles are observed.

Klos Dehring et al. found that the gene for xCCDC78 is highly up-regulated in ciliated epithelia of *Xenopus* embryonic skin. Using immunofluorescence and fluorescently tagged CCDC78 in MTECs, the authors confirmed the localization of CCDC78 at the acentriole foci (deuterosomes) Inhibiting CCDC78 resulted into complete loss of CEP152 to these acentriole foci, suggesting that CCDC78 plays an important role in recruiting CEP152 at deuterosomes. Interestingly, the depletion of xCCDC78 did not result in complete blocking of centriole amplification, which in turn suggests the presence of some yet to be identified functional hierarchy between Deup1 and CCDC78. 

Al Jord et al. [28] used transgenic mice expressing green fluorescent protein (GFP)-tagged Centrin2 (core centriole protein, Cen2-GFP) in ependymal cells and reported four stages of centriole amplification using live cell imaging (Figure 2b). They were able to identify similar stages as reported in MTECs by Zhao et al. [25]. The four main stages are: Stage I, where Cen2-GFP rings or “halos” were observed accumulating in the cytoplasm. In stage II, the Cen2-GFP fluorescence signal was intensified and formed a flower-like arrangement. In stage III, the Cen2-GFP signal was dispersed just like a flower-dissociation. Finally, in the stage IV, the Cen2-GFP^+^ centrioles migrated towards the apical membrane and initiated the extension of motile ciliary tufts. The authors used correlative images of 3D-SIM and transmission electron microscopy (TEM) to confirm that the Cen2-GFP halos were organized around the deuterosome structures. Furthermore, serial EM sections of centrosomes in early differentiating ependymal progenitors showed that early deuterosomes are positioned adjacent to the daughter centriole at its proximal end. In other words, the deuterosome could just be considered an intermediate stage, directed by the daughter centriole to amplify centrioles during ciliogenesis. They also reported that immediately after the release of the last halo/deuterosome from the daughter centriole, up to five procentrioles were also visible directly around the proximal portion of the pre-existing centrioles. The halo stage is also found to be positive for early centriole markers (PLK4, CEP152, SAS6, STIL, CPAP/CENPJ, CEP120, CP110) and the flower stage is positive for late centriole markers (POC5, glutamylated tubulin). This work establishes a good correlation between the pre-existing centriole and deuterosomes organization in ependymal cells.

Mori et al. [84] showed that the transcription factor E2f4 could translocate from the nucleus to the cytoplasm at stage II (ALI 0–2 days) of centriole biogenesis in airway epithelial progenitor cells. 3D-SIM images revealed that the cytoplasmic E2f4 is localized at the procentrioles generated via the centriole- and as well as deuterosome-dependent pathways. It is also known that during ciliogenesis, the transcription factor E2f4 is required for the deuterosome assembly by altering the activation of a broad regulatory protein Multicilin [85]. Accordingly, in the lentivirus *E2f4^WT^*-transduced cells accumulation of apical Deup1^+^ granules were observed by Mori et al., whereas the transcriptionally inactive *E2f4^∆DBD^*-transduced cells showed no detectable signal for Deup1. Moreover, preventing the cytoplasmic localization of E2f4 (E2f4^∆NES^) resulted in severely affected Deup1^+^ apical cytoplasmic aggregates. This study highlights the importance of both the nuclear and cytoplasmic pools of E2f4 in regulating centriole biogenesis in MCCs. Interestingly, the cytoplasmic pools of E2f4 were reported near the pre-existing centrioles during the early stages of centriole biogenesis in these airway epithelial cells.

Overall, several reports corroborate that the early stages of centriole biogenesis in MCCs occurs near the pre-existing centrioles.

## 6. Conclusions and Perspective

In summary, a new picture of the deuterosome-dependent centriole amplification pathway in MCCs is emerging according to which centriole amplification can be sub-divided into the following five major stages: (1) centrosome stage: it refers to the classic arrangement of centrioles in a centrosome; (2) halo stage: procentrioles are visible around the deuterosomes growing adjacent to the daughter centriole; (3) flower stage: the procentrioles grow/mature on the deuterosomes and simultaneously both the mother and daughter centrioles organize rosettes of procentrioles; (4) flower dissociation stage: centrioles get dissociated from their respective parent, that is either pre-existing centrioles or deuterosomes; and (5) apical migration: centrioles migrate towards the apical membrane and initiate ciliary tuft formation.

Very recently, Zhao et al. [86] investigated the requirement of mother centrioles for deuterosome organization in the mouse tracheal epithelial and ependymal cells. In these cells, the authors depleted PLK4 by shRNA which inhibited any new centriole formation. Surprisingly, they observed that PLK4 depletion resulted in only 21% reduction in average deuterosome numbers per cell as compared to the control cells. It has been observed in the cycling cells that ablating parent centrioles triggers centriole biogenesis via the de novo pathway [21,22]. It can be anticipated that a similar de novo pathway might also reinforce deuterosome organization in the PLK4 depleted MCCs which lack pre-existing centrioles. Nevertheless, an explicit understanding of the interplay between de novo and centriole-dependent pathways can only be achieved by further investigations depicting the role of pre-existing centrioles in the deuterosome organization.

There are several obvious questions that also need to be addressed in the future. For instance, independent reports have shown that the deuterosome specific proteins Deup1 and CCDC78 could interact with Cep152, but it would be interesting to explore the details of these interactions. The deuterosome specific markers Deup1 and CCDC78 have different effects on centriole amplification pathways upon inhibition, which indicates a yet to be identified functional hierarchy at deuterosomes. Since the identity of some of the specific deuterosome markers is already known, this can be used to carry out proteomic studies in order to identify novel molecular players. It would also be tempting to explore the role of cell cycle regulators during the amplification stages. In this regard, Cyclin O (CCNO) is found to be specifically expressed in MCCs and its expression peaks during the earliest phase of deuterosome formation. Interestingly, there is a report in which a mutation in the *CCNO* gene was found to affect cilia number in MCCs which in turn resulted in airway diseases in humans [27]. The mechanisms regulating transcription and translation during deuterosomes organization are also largely unknown. Recent reports have identified the involvement of a transcription factor MULTICILIN (Mcidas), as a direct and broad regulator of transcriptional programs responsible for DD-ciliogenesis in MCCs [87]. Multicilin was also found to regulate the transcription of *CCNO* [88]. Additionally, Multicilin can form a complex with the family of transcription factors, E2F. Interestingly, it has been shown that fusing activated E2F4VP16 form at the C-terminus of Multicilin results in massive centriole duplication via the deuterosome pathway in primary mouse embryonic fibroblasts. This suggests that modulating the transcription function of Multicilin could contribute to the centriole biogenesis in differentiating MCCs [85]. Another protein GEMC1 (GMNC) with similar domain organization as Multicilin has been reported to be a critical regulator of MCC differentiation in mice [89]. Importantly, the function of GMNC in MCC differentiation is found to be evolutionarily conserved [90]. The current literature in the field has opened a new gateway for further studies which will be required for a better understanding of functional modules during centriole amplification in MCCs. Such studies are required for shedding more light on the etiology of ciliopathies that result due to centriole biogenesis defects in MCCs.
